# Fatigue Failure Analyses on a Ti-45Al-8Nb-0.2W-0.2B-0.1Y Alloy at Different Temperatures

**DOI:** 10.3390/ma5112280

**Published:** 2012-11-09

**Authors:** Xi-Shu Wang, Min Zhang, Xi-Ping Song, Su Jia, Qiang Chen, Norio Kawagoishi

**Affiliations:** 1 Department of Engineering Mechanics, AML, Tsinghua University, Beijing 100084, China; E-Mail: jia-s11@mails.tsinghua.edu.cn; 2 State Key Laboratory for Advanced Metals and Materials, University of Science and Technology Beijing, Beijing 100083, China; E-Mails: zm-821202@163.com (M.Z); xpsong@skl.ustb.edu.cn (X.-P.S); 3 Division of International Exchange Support, Center for Globalization, Kumamoto University, Kumamoto 860-8555, Japan; E-Mail: qchen@kumamoto-u.ac.jp; 4 Department of Mechanical System Engineering, Daiichi Institute of Technology, Kagoshima 899-4395, Japan; E-Mail: n-kawagoishi@daiichi-koudai.ac.jp

**Keywords:** titanium aluminum, fatigue crack, microstructure, *in situ* SEM test

## Abstract

The fatigue micro crack initiation and propagation tests of a TiAl alloy with 8% Nb content were carried out by using scanning electron microscopy *in situ* technology at room temperature and at 750 °C. These results indicated that the fatigue micro crack initiation was mainly caused by the stress concentration at room temperature, but at an elevated temperature (750 °C) the multi-cracks were caused by the coupled factors of both lamellar microstructure and stress concentration. Therefore, fatigue micro crack initiation behavior is much more dependent on the lamellar structure at an elevated temperature. One of the reasons is that the elevated temperature degrades the interface strength between the lamellar of the TiAl alloy with 8% Nb content. Therefore, the small fatigue crack propagation behavior of the alloy exhibited a mixture damage model of interlamellar and translamellar at a micro scale. The crack growth path and fracture characteristics provided a proof of crack deflection, branching and/or bridging induced either by interlamellar or by translamellar failure mode.

## 1. Introduction

TiAl alloys now appear as potential competitors in jet engines and turbines at elevated temperatures. Some excellent properties of a high Nb element addition into TiAl alloy at the elevated temperature have been recognized [1,2,3]. The TiAl alloy with 8% Nb content has near lamellar (NL) microstructure based on the arc melting method. Fracture behavior of the general TiAl alloys has been intensively investigated over the past 20 years. For example, Kim *et al.* [4] described that the fracture behavior in duplex phase TiAl alloys at room temperature (RT) were dominated by the transgranular cleavage-like failure and often exhibited river patterns. The lamellar specimens of TiAl alloys exhibit three characteristic fracture modes at RT: translamellar, interlamellar and mixture fracture modes, which depend on the lamellar orientation tilted to the crack path or principal stress axis. Inui *et al.* [5] investigated the tensile deformation of polysynthetically twinned crystals of TiAl alloy and found that the mechanical parameters such as the elongation ratio and yield stress are strongly dependent on the inclined angle between the lamellar interfaces and tensile axis. Cao *et al.* [6,7,8] reported that the crack initiation occurred mostly at grain boundaries and/or interfaces between the lamellar of TiAl alloys under various loading modes based on the fracture cross-section analysis method. Their results indicated that the fracture characteristics of TiAl alloys under various loading modes depended on the number of cycles such as low-cycle fatigue (LCF, N < 10^4^) and high-cycle fatigue (HCF, N = 10^4^–10^6^), especially giga-cycle fatigue (GCF, N > 10^7^). Since the fatigue life of components and structures of TiAl alloy is mainly controlled by the fatigue crack initiation and propagation lives, most literature indicated recently that the LCF crack initiation life at RT of general TiAl alloys depends strongly on the microstructure and experimental conditions including the defects, stress ratio as well as mechanical properties (such as the ductility, fracture strength, fracture toughness *etc*.) [1,9,10,11,12,13,14]. In addition, Ruppen *et al.* [15] and Mendez *et al.* [16], respectively, reported the temperature and environmental effects on fatigue damage behavior of titanium alloys; Bridier [17] reported the slip and fatigue crack formation process in a α/β titanium alloy in relation to crystallographic texture on different scales and Pilchak [18] reported the crystallography of fatigue crack initiation and growth in fully lamellar Ti6Al4V. However, most literature can not relate to the HCF failure analysis of TiAl alloy with high Nb content. Therefore, the fatigue crack initiation behavior of TiAl alloy should be clarified for the reliable design of machine components and structures. However, as the HCF issues regarding the introduction of high Nb element into TiAl alloy, the micro cracking behavior and elevated temperature effects were seldom reported in the past decennium, especially by the method of detecting the fatigue surface micro crack initiation and propagation behavior by using *in situ* SEM observation technology. For most metals, the HCF fatigue damage arising from the accumulatively irreversible plastic deformation at the crack tip is much more difficult to be measured in micro scale than that in LCF. These research results of LCF crack initiation and propagation behavior at RT cannot sufficiently reflect the governing mechanism in HCF situation of TiAl at different elevated temperatures, especially TiAl alloy with high Nb content [1,2,3]. Therefore, to optimize the microstructure and improve HCF resistance of the TiAl alloy with high Nb content, an *in situ* SEM investigation should be conducted [19,20,21,22,23,24,25,26,27]. In order to fill the gap between the understanding of the microstructure and fatigue cracking mechanism of TiAl alloys with high Nb content, and providing a criterion to evaluate next-generation crack formation models at different experimental conditions, this paper mainly investigates fatigue failure characteristics of TiAl alloys with high Nb content based on free surface crack detection by using *in situ* SEM technology and fracture surface analysis, including special attention paid to the influence of parameters such as lamellar microstructure and temperature effect.

## 2. Experimental Method and Material

**Figure 1:**
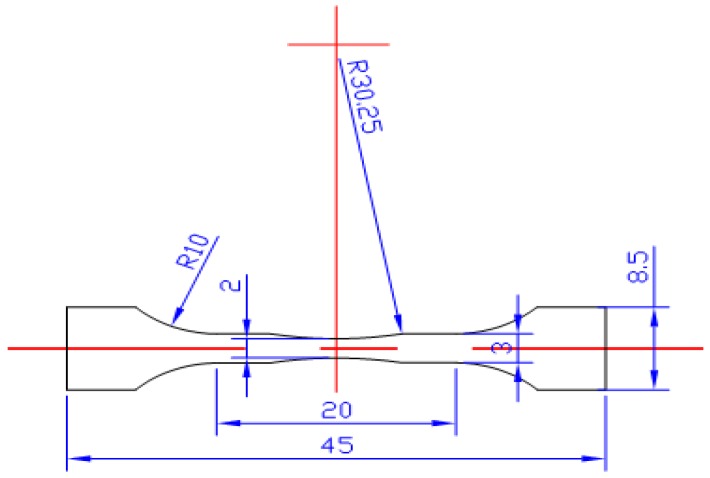
Dimension of specimens for *in situ* SEM observation tests (the thickness of the samples is about 0.7–1.0 mm, and all dimensions unit: mm).

**Figure 2:**
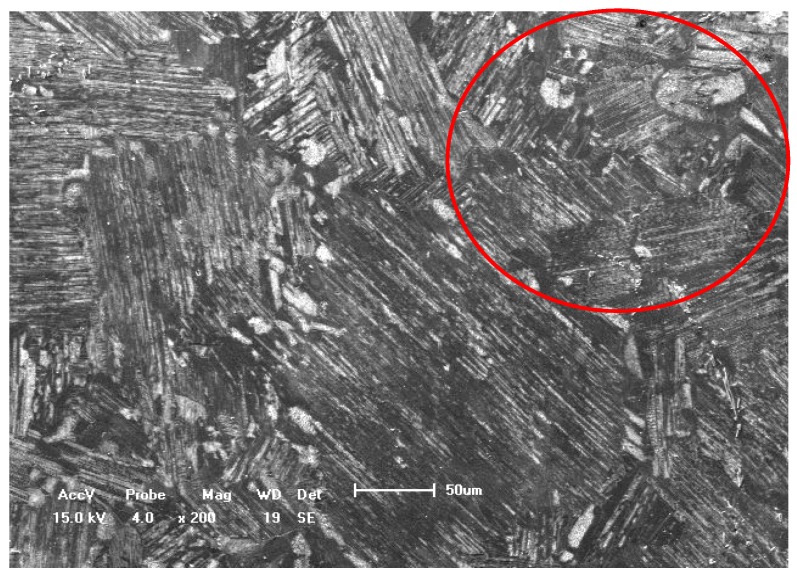
Surface morphology of TiAl alloy with 8% Nb content (bar scale 50 μm).

The material used in this work is TiAl with 8% Nb content based on the arc melting method. The specimen was cut into a dog-bone shape directly from an ingot by wire cutting electrical discharge machining, as shown in Figure 1. Due to the fact that the microstructure of this alloy is similar to a stochastic distribution of lamellar colony, which means the colony size is about 70 μm [3], the orientation of specimen’s axis was randomly cut. Therefore, it can be approximately assumed as an isotropic material in macro scale as shown in Figure 2. The main mechanical properties were listed as follows: 0.2% yield strength are 617 MPa and 586 MPa, tensile strength are 690 MPa and 795 MPa and elongation are 0.50%–0.78% [2,3] and 1.10% at room temperature and 750 °C, respectively. According to the literature reported previously [1,2,3], the mechanical properties of TiAl with high Nb content at room and different elevated temperatures are different. For example, with the increase of temperature, the ultimate tensile strength (UTS) and elongation (δ%) increase, but the yield strength (YS) is basically invariant. The Young’s modulus is about 180 GPa and the Poisson’s ratio is about 0.25 [3,28]. The viewing surface of all specimens was carefully polished into a mirror surface. A single notch with the radius of about 80 μm and the depth of about 300 μm was manually created at the center of the specimen in order to generate a local stress concentration region [21,26,29].

The fatigue crack initiation and propagation tests, referred to in previous literature [19,20,21,22,23,24,25,26,27,29,30], were carried out in the vacuum chamber (10^−4^ Pa) of SEM by using a specially designed servo-hydraulic testing system. The testing system can provide pulsating loads (sine wave) at 10 Hz, a maximum capacity of ±1 kN and a displacement range of ±25 mm. All fatigue cracking tests were taken at a stress ratio of *R* = 0.1 and maximum stress of 345 MPa and 395 MPa at the different temperatures, respectively. The signal of the SEM was directly transferred to a computer via a direct memory access type A/D converter, making it possible to sample 960×1280 frames of SEM images. The surface length of the small fatigue crack was measured directly by the calibrated scale of the SEM system. These HCF crack lengths were measured in the projection of the loading direction even if the crack occurred in deflection in the propagation direction of crack, and at maximum open state (*R* = 0.1) under *in situ* SEM observation processing. The elevated temperature was controlled by a DC powered thermoelectric couple, whose control accuracy is about ±2 °C within 800 °C [23,29]. To accelerate the fatigue damage of the specimen, the pulsating loads were applied in the frequency of 8 Hz. But when the cracks were observed, the loading frequency was reduced to 0.05–0.10 Hz to record the initiation and propagation process of fatigue micro cracks clearly.

## 3. Results and Discussion

### 3.1. Surface Fatigue Crack Initiation and Propagation Behavior

**Figure 3:**
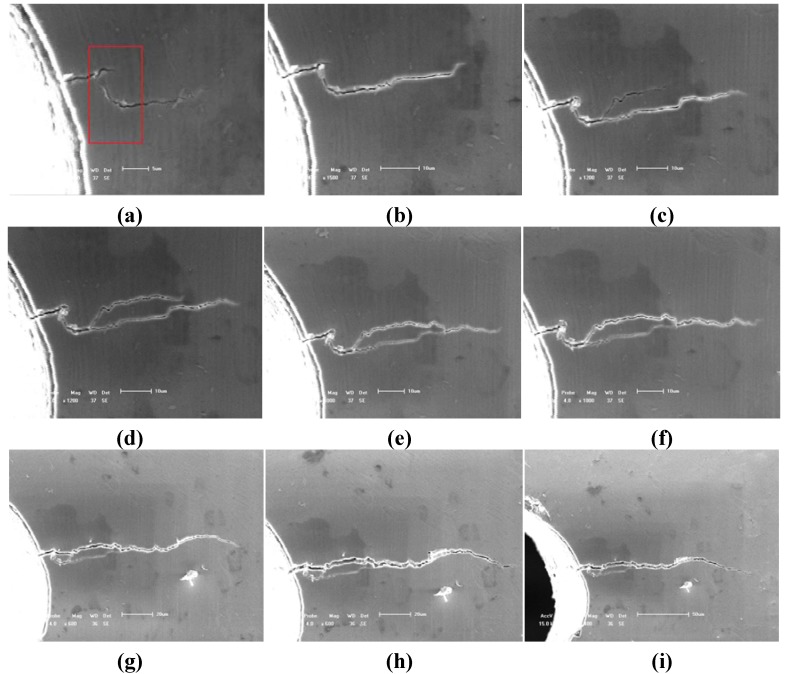
Fatigue crack propagation behavior of TiAl alloy with 8% Nb content at room temperature under different cycles of the same applied stress of 345 MPa. (**a**) N = 16,400, (bar scale 5 μm); (**b**) N = 31,400, (bar scale 10 μm); (**c**) N = 45,604, (bar scale 10μm); (**d**) N = 47,226, (bar scale 10 μm); (**e**) N = 55,548, (bar scale 10 μm); (**f**) N = 66,309, (bar scale 10 μm); (**g**) N = 76,120, (bar scale 20 μm); (**h**) N = 76,356, (bar scale 20 μm); (**i**) N = 76,459, (bar scale 50 μm).

Due to the lower elongation (<2%) and lower fracture toughness of TiAl alloy with 8% Nb content, the fatigue life of this alloy is mainly controlled by the initiation and propagation lives of micro cracks although it is rather difficult. Figure 3 illustrates a typical case of HCF micro crack initiation behavior and early stages of propagation behavior of TiAl alloy with 8% Nb content after different number of cycles (from N = 1.64 × 10^4^ to 7.65 × 10^4^) at RT. The *in situ* SEM images indicate that the small fatigue crack initiation occurred at the immediate vicinity of the root of notch, which is the result of the stress concentration, as shown in Figure 3a. The small fatigue crack caused by the stress concentration is about 8 μm long and the small fatigue crack caused by lamellar colony is about 5 μm with an angle tilted to the applied loading direction after 1,6400 cycles as shown with a rectangle mark in Figure 3a. This is because the small fatigue crack initiation behavior for most of the alloys depends strongly on the defects and microstructure characteristics [23,24,25,26]. With increasing of the cyclic numbers (from N = 1.64 × 10^4^ to 7.65 × 10^4^) under the same applied loading, the small fatigue crack (whose length is over than 70 μm) propagated gradually perpendicular to the applied loading direction as shown in Figures 3b–i. Due the fatigue crack tips (caused by the effect of stress concentration and lamellar colony interfaces) are not in the same stress level among the local regions, the multi-cracks may occur easily in deflection, branching and/or bridging ways as shown in Figure 3g. Therefore, the early stage of fatigue crack propagation behavior is slightly different from the fatigue fracture crack propagation behavior, as shown in Figure 3h-3i. The early stage of fatigue crack propagation behavior of the TiAl alloy with high Nb content at RT exhibits a typical evidence of micro crack deflecting, branching and/or bridging. At the same time, the fatigue crack propagation behavior in the macro scale (which is over 150 μm) is slightly different from that in micro scale (which is defined to be less than 70 μm).

**Figure 4:**
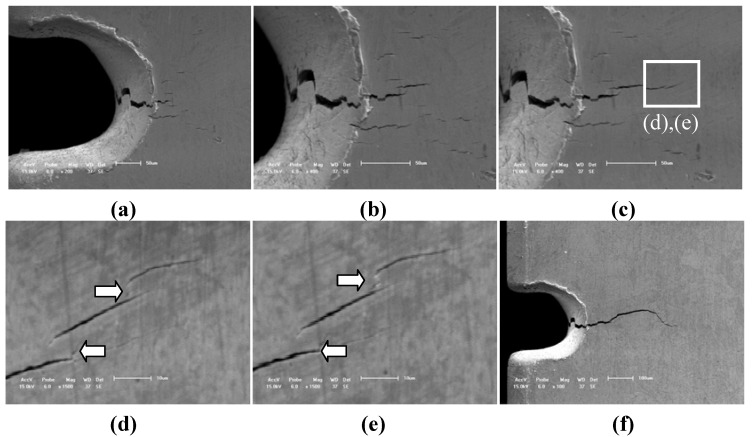
Fatigue crack propagation behavior of TiAl alloy with 8% Nb content at 750 °C under different cycles of the same applied stress of about 395 MPa. (**a**) N = 27,354, (bar scale 50 μm); (**b**) N = 27,482, (bar scale 50 μm); (**c**) N = 27,892, (bar scale 50 μm); (**d**) N = 70,471, (bar scale 10 μm); (**e**) N = 81,865, (bar scale 10 μm); (**f**) N = 85,870, (bar scale 100 μm).

Figure 4 illustrates another typical case of HCF crack initiation and propagation behavior of TiAl alloy with 8% Nb content at 750 °C. Compared with the fatigue crack initiation behavior at RT, the fatigue micro crack also occurred at the root of notch after 27,354 cycles but more micro cracks were induced by lamellar colony in the close notch region. Therefore, the effect of the elevated temperature of 750 °C on the HCF crack initiation behavior cannot be ignored. These micro cracks are dominated not only by the stress concentration, the shape and size of the lamellar colony as shown in Figures 4a–c but also by the elevated temperature. The main action of the elevated temperature is to decrease the interface strength of lamellar colony and the lamellar interface strength. For example, when the cycle number reaches 27,892, the multi-cracks gradually formed a main fatigue crack length of about 100 μm, as shown in Figure 4c. However, the multi-cracks initiated randomly at lamellar interfaces or lamellar colony interface, and the parallel space between cracks is approximately from 3 μm to 5 μm, which are similar to the results in previous literature [1,7,8,9]. However, the HCF crack initiation behavior (within 100 μm) at the elevated temperature of 750 °C was obviously different from that at RT as shown in Figures 4d–e and Figure 3a. With the increase of cycle number, the parallel multi-cracks in the micro scale (over than 100 μm) were gradually deflecting, branching (as shown by arrow marks in Figure 4d,e) and bridging to a main fatigue crack as shown in Figure 4f. Due to the lamellar microstructure feature of TiAl alloy with 8% Nb content, the early stage of fatigue crack propagation occurs intermittently rather than continuously in lamellar or lamellar colony interfaces at elevated temperature, and HCF crack initiation tended to occur mostly at the parallel mode in the lamellar or lamellar colony interfaces. Therefore, there are much more branching cracks, deflection cracks or bridge linking cracks in micro scale at elevated temperatures than that at RT (as shown by arrow marks in Figure 4d,e).

### 3.2. Fatigue Fracture Analysis at Different Temperatures Based on Cross-Sections

**Figure 5:**
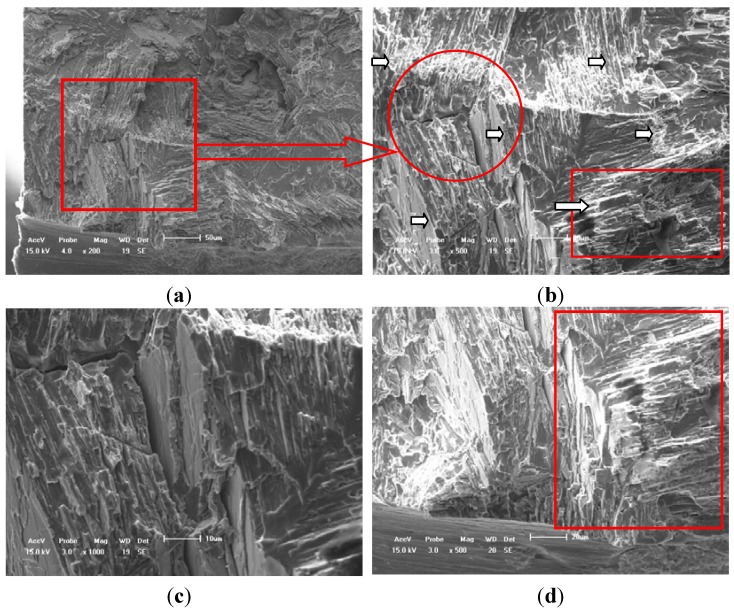
Fatigue fracture characteristics of TiAl alloy with 8% Nb content at room temperature. (**a**) Facet surface, (bar scale 50 μm); (**b**) Translamellar and interlamellar decohesions, (bar scale 20 μm); (**c**) Translamellar decohesion, (bar scale 10 μm); (**d**) Interlamellar decohesion, (bar scale 20 μm).

The SEM observation of fatigue fracture surface greatly assists understanding of the failure process of TiAl alloy with 8% Nb content when it was combined with the free surface fatigue crack initiation and propagation behavior by using *in situ* SEM observation tests. On the other hand, as these samples used in HCF crack propagation tests are without any heat treatment, there seldom exists any metallurgy element-rich area compared with literature [1,6], in which the area was defined as the original position causing the fatigue crack initiation. Figure 5 illustrates the fatigue fracture characteristic of a typical case at RT. The fatigue crack initiation was mainly caused by the notch effect. In many fatigue fracture cases, a crucial reason or mechanism of HCF crack initiation was caused by the surface conditions of specimen such as oxidized layer, machining flaws or coarse lamellar colonies perpendicular to the applied load axis, which extremely shorten the forming time of the HCF crack as shown in Figure 3a and Figure 5b-5d. In addition, the concave-convex crack deflection exists not only in arrowhead direction but also on through the thickness of fracture surface as shown in Figure 5b.

**Figure 6:**
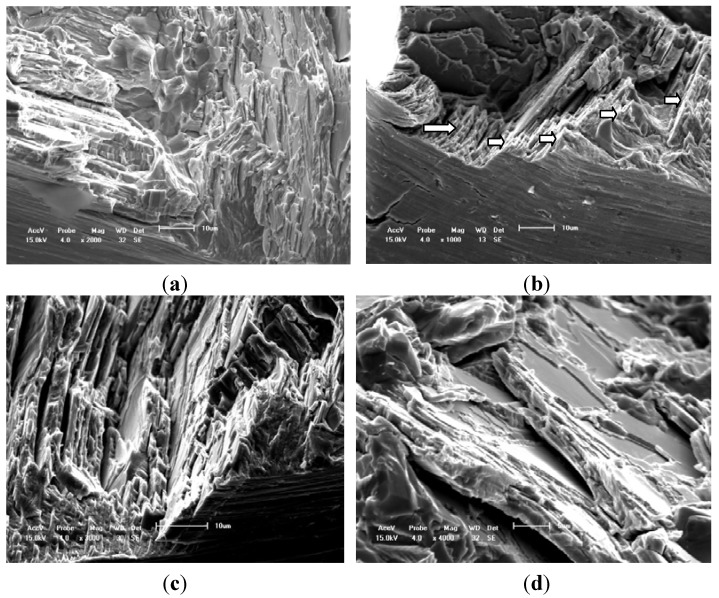
Fatigue fracture characteristics of TiAl alloy with 8% Nb content at 750 °C. (**a**) Scale bar 10 μm; (**b**) Scale bar 10 μm; (**c**) Scale bar 10 μm; (**d**) Scale bar 5 μm (Interlamellar decohesion).

Figure 6 illustrates the fatigue fracture characteristic of a typical case at 750 °C. These SEM morphologies of fracture surface at elevated temperature reflect the fact that the fatigue fracture is complex to model in the early stage of crack propagation. The fracture behavior is mainly caused by the interlamellar and translamellar fracture modes, and which is different from the simple mode I fatigue crack propagation direction in micro scale (see the white arrow marks) after comparing with the results shown in Figure 5 and Figure 6. In addition, there are many more interface cracks in the interlamellar colony at 750 °C than that at RT as shown in Figure 5c and Figure 6c, which can support the conclusion from *in situ* SEM observation. The secondary crack along the lamellar colony boundaries and the interface crack among the lamellar colony are clearly found. Therefore, the fatigue fracture characteristics at an elevated temperature are slightly different from the reported results at LCF in previously literature [1,2,6,7,8,9,10,11,12,13]. Moreover, the zigzag morphology regions in HCF fracture surface of literature are much larger than that in tensile fracture surface [1,2,6] (seldom saw the river pattern fatigue characteristic). The length of the zigzag morphology region is about 80–100 μm, which is slightly larger than the average size of lamellar colony of this alloy as shown in the circled region of Figure 2. In addition, comparing with the difference of fatigue fracture mode of literature between the room and elevated temperature, the HCF fracture probability of interlamellar failure region at elevated temperature is larger than that at RT and there are many more interface cracks within the lamellar colony at elevated temperature than that at RT.

### 3.3. HCF Fracture Model of Tial Alloy With 8% Nb Content at Different Temperatures

**Figure 7:**
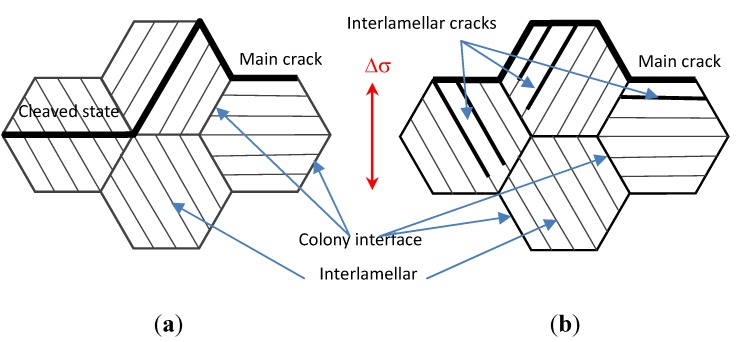
Fatigue fracture modeling of TiAl alloy with 8% Nb content [10]. (**a**) At room temperature; (**b**) At elevated temperature.

Figure 7 illustrates the differences of HCF micro crack propagation mode and lamellar colony misorientation at RT and 750 °C. The HCF crack propagated mostly along the projection of the applied load axis at RT so that there are major cracks of the cleaved lamellar colony as shown in Figure 7a. However, HCF crack propagation at elevated temperature often selects to link some of the pre-existing micro cracks within the lamellar colony in which there are many more interface cracks, whose propagation model induces more deflections, ligament bridging in the total fatigue crack propagation process as shown in Figure 7b. This is because the fatigue crack propagation resistance is attributed the high fraction of misorientated crack growth. In short, less deflection, branching, and ligament bridging phenomena of fatigue crack propagation at RT resulted in weak incompatibility of plastic deformation among adjacent lamellar colonies. Therefore, the fatigue crack propagation resistance at RT decreased [31] compared with that at the elevated temperature. Therefore, the high content of the Nb element added into the TiAl alloy indicated an improvement in the crack propagation resistance at the elevated temperature, especially in the early stage of fatigue crack growth resistance in 10^7^–10^8^ cycles [32]. Another point is that the fracture toughness and elongation of TiAl alloy with 8% Nb content at the elevated temperature is better than that at RT so that the fatigue crack initiation and early stage of fatigue crack propagation resistance are improved at elevated temperature by the additional higher Nb content in the TiAl alloy.

## 4. Conclusions

The fatigue crack initiation of TiAl alloys with 8% Nb content strongly depends on the surface defects, such as notches. The effect of an elevated temperature on the fatigue crack initiation behavior mainly exhibits the phenomenon that there are many more interface cracks than that at room temperature, as occurred in the lamellar colony. Therefore, there is much more micro crack branching, deflection and bridge linking in the immediate vicinity of the lamellar colony at elevated temperatures than that at RT. One of main reasons, is that the elevated temperature decreased the interface strength in the lamellar colony and between the interlamellar structure, but the HCF crack propagation resistance at the elevated temperature is greater than that at RT, especially the early stage of fatigue crack propagation of TiAl alloy with 8% Nb content.

The early stage (80–100 μm) of fatigue crack propagation behavior of TiAl alloys with 8% Nb content mainly exhibits many more crack deflection behavior, which is not only along the perpendicularly applied loading direction but also on through the thickness of the sample. Furthermore, the effect of temperature on the HCF crack propagation behavior of TiAl alloys with 8% Nb content indicates that the probability of many more cracks deflection and interlamellar cracks at the elevated temperature is greater than that at RT. Therefore, the fatigue crack propagation model at elevated temperatures should be a mixed crack propagation model. That is, the fatigue micro crack propagation model at the elevated temperature is not a fracture model when the crack length is less than 100 μm. This is because of the 750 °C decreases in the interface strength of the lamellar colony and interlamellar strength, so that the HCF crack occurs early in these regions. However, the macro crack propagation resistance at the elevated temperature is greater than that at RT for TiAl alloy with 8% Nb content.

The HCF fracture characteristics of TiAl alloy with 8% Nb content are slightly different from that of the LCF or tensile fracture surface of general TiAl alloys due to the effect of fatigue crack propagation path. The former characteristic is that there are many more zigzag cracks and the latter is that there are river pattern fatigue characteristics. It is reasonable to investigate the fatigue damage mechanism of alloys by combining the analyses of *in situ* SEM observation and fracture surface, especially in the meso-scale.
